# Enantio and Diastereoselective Addition of Phenylacetylene to Racemic *α*-chloroketones

**DOI:** 10.3390/molecules16065298

**Published:** 2011-06-23

**Authors:** Silvia Alesi, Enrico Emer, Montse Guiteras Capdevila, Diego Petruzziello, Andrea Gualandi, Pier Giorgio Cozzi

**Affiliations:** Alma Mater Studiorum, Dipartimento di Chimica “G: Ciamician”, Università di Bologna, Via Selmi 2, 40126 Bologna, Italy

**Keywords:** alkynylation, (*R*,*R*)-salen, chloroketones, Me_2_Zn, phenylacetylene

## Abstract

In this report, we have presented the first diastereoselective addition of phenylacetylene to chiral racemic chloroketones. The addition is controlled by the reactivity of the chloroketones that allowed the stereoselective reaction to be performed at –20 °C. Chiral racemic chloroketones are used in the reaction. By carefully controlling the temperature and the reaction time we were able to isolate the corresponding products in moderate yields and with good, simple and predictable facial stereoselection. Our reaction is a rare example of the use of chiral ketones in an enantioselective alkynylation reaction and opens new perspectives for the formation of chiral quaternary stereocenters.

## 1. Introduction

**Scheme 1:**
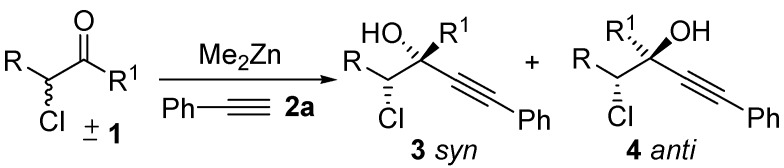
Addition of a phenylacetylene to racemic chloroketones.

The addition of carbon nucleophiles to reactive electrophilic functions, such as C=O and C=N double bonds, is a process of fundamental importance in the development of chemical synthesis [1,2]. Among the various nucleophilic species available, alkynes are excellent reagents for mild and selective C-C bond forming reactions [3,4,5]. In 2005, we found that mixtures of Me_2_Zn and acetylenes are able to promote the room temperature alkynylation of aldehydes, ketones and imines to furnish propargylic alcohols in good to excellent yields [6]. We have also developed an enantioselective addition of phenylacetylene to ketones based on this concept [7]. Since our report, the enantioselective alkynylation of ketones using R_2_Zn as deprotonating agent in the presence of chiral ligands has been the subject of a number of interesting studies [8,9,10,11,12,13]. However, the diastereoselective and enantioselective addition of acetylides has never been investigated in the case of ketones. Recently, organocatalytic reactions have made possible the simple preparation of optically active *α*-chloro- or *α*-bromoketones, useful starting materials for the preparation of densely functionalized building blocks [14]. However, these useful starting material are difficult to isolate and the subsequent reaction needs to be performed *in situ*. On the other hand, racemic *α*-haloketones are inexpensive and readily accessible reagents. If the addition of a nucleophile to a racemic haloketone is realized in the presence of a chiral catalyst in a stereoselective manner, highly densely functionalized building blocks containing a quaternary stereocenter could be prepared (Scheme 1). 

Herein, we report a successful realization of this concept using the Zn(Salen) promoted addition of acetylene to racemic *α*-chloroketones.

## 2. Results and Discussion

**Scheme 2:**
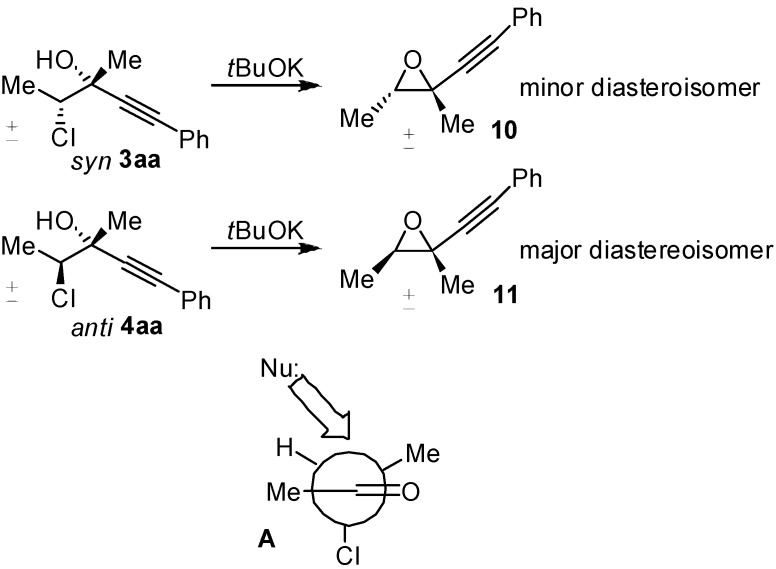
Assignment of the relative configuration of the diastereoisomers obtained by addition of phenylacetylene to a racemic chloroketone.

During our studies in the addition of phenylacetylene promoted by Me_2_Zn performed in the absence of air we found that *α*-chloroketones were particularly reactive substrates, and the reaction of 3-chloro-2-butanone with phenylacetylene in the presence of Me_2_Zn furnished the corresponding alcohol quantitatively, albeit as a mixture of diastereomers in 52:48 ratio (Scheme 1). In order to improve the diastereoselection we have investigated the reaction in the presence of different ligands **5-9**. We have found that a good dr was obtained when the reaction was performed in the presence of the racemic Salen ligand **8**. On the other hand, other Schiff bases were also able to increase the dr of the reaction. The possibility to use *α*-chloroketones as substrates for the addition of nucleophiles takes advantage of their enhanced reactivity, and this opens new possibility for stereoselective addition of nucleophiles in organic synthesis [15]. As the reactivity of the chloroketones, compared to other ketones, is remarkable (even in the presence of the Salen ligands), we decided to investigate the reaction performing the addition of phenylacetylene in the presence of enantiopure Salen ligands. We have disclosed the first addition of phenylacetylene to ketones controlled by the Salen ligand [7], and other ligands able to promote the addition of alkyne to ketones were introduced by other groups [8,9,10,11,12,13]. The addition of phenylacetylene to silylketones promoted by Salen ligands was reported by Chan and Lu [16]. In all these reports it is clearly shown that the reactivity of the ketones is quite different compared to aldehydes and in all the systems reported, long reaction times and a high catalyst loading are required for good stereoselection. The simple stereoselection of the reaction was assigned as *anti* by chemical correlation with the diastereoisomeric epoxides **10** (Scheme 2).

**Table 1:** Diastereoselective addition of phenylacetylene in the presence of ligands.

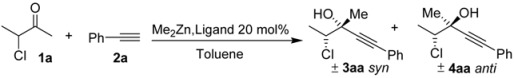			
Entry ^a^	ligand	*syn*%	*anti*%
1 ^b^	--	48	52
2	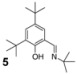	23	77
3	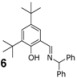	29	71
4	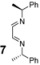	23	77
5	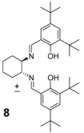	22	78
6	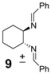	29	71
7 ^c^	--	6	94

^a^ All the reactions were performed at rt under nitrogen atmosphere Me_2_Zn (3 equiv.) and phenylacetylene (3 equiv.) were added to toluene in a flask under strictly anhydrous conditions, and stirred 10 min, then the ligand (20 mol%) was added and the reaction mixture and stirred for 5–10 min. Finally, the chloroketone (1 equiv.) was added. The reaction was stirred under completion (monitored by TLC, 16–24 h) and quenched with water. The dr was determined on the crude reaction mixture by ^1^H-NMR. ^b^ The reaction was performed in the presence of Me_2_Zn without ligands. ^c^ The reaction was performed with lithium phenylacetylide, prepared by the addition of 1 equiv. of *n*-BuLi to 1.1 equiv. of phenylacetylene at 0 °C.

The mixture of the diasteroisomers was transformed to the epoxides by treatment with *t*BuOK in THF at –20 °C. The ^13^C- and ^1^H-NMR data of the mixture of isolated epoxides were compared with literature data [17]. It is worth adding that the reaction of the lithium acetylide to the chloroketone furnished the desired product with high stereoselectivity (Table 1, entry 7). The major diastereoisomer derived by the attack of the alkyne to the chiral chloroketone can be rationalized by invoking the Felkin-Anh model [18,19,20,21,22]. While the reaction with Me_2_Zn afforded the desired products with low stereoselectivity (Table 1, entry 1), the the Zn(Salen) formed *in situ* controls the stereoselection through the complexation of the chloroketone to the zinc Lewis acidic center. The simple *anti* diastereoselection obtained in the case lithium phenylacetylene and with ligand mediated addition of zinc phenylacetylide can be rationalized by a Felkin-Anh transition state in which the nucleophilic alkyne attaches the carbonyl group in a conformation in which the chlorine is the larger group (Scheme 2, Figure A).

The preliminary results obtained with **1a** gave us the indication that the reactivity of the chloroketone was very high, and that it was possible to reduce the temperature for the stereoselective reaction. It is noteworthy that the Salen catalyzed addition of phenylacetylene to ketone is performed at rt and no addition occurs with aliphatic or aromatic ketones at lower temperatures (0 °C or below). The increased reactivity of chloroketone thus allowed the investigation of the reaction at reduced temperature. Using commercially available racemic 3-chlorobutanone (**1a**) as a model substrate, we have performed the reaction at low temperature, in the presence of the (*R*,*R*)-Salen **8** and in Table 2 we report the results of this investigation. The reaction is stereoselective and the corresponding adducts can be isolated in high enantiomeric and good diastereoisomeric excesses, albeit in low to moderate yield. The enantiomeric excess obtained in the reaction is a function of the conversion. In fact, in order to keep the stereoselection very high it is important to stop the reaction after 60 hours at –20 °C; if the reaction is conducted at 0 °C, the adduct is isolated with a dr of 4:1 in favor of the *anti* diastereoisomer in good yield (60–70%) but in very low ee. If the reaction is not stopped at –20 °C after 60 h, the conversion is increased and the yield can be higher than 50%, but the facial stereoselection of the isolated diastereoisomers was quite low. Performing the reaction at increased temperature (>0 °C) the *syn* and *anti* stereoisomers are again isolated with good yield but in very low enantiomeric excess. This is straightforwardly explained by considering the reactivity of the chloroketone and the background reaction. At room temperature the Me_2_Zn promoted addition of phenylacetylene to ketone is occurring without the catalysis of the Zn(Salen) complexes [6]. The conversion and the isolated yield of the products can be improved at the expense of the enantiomeric excess. In order to increase the reaction rate of the reaction the excess of Me_2_Zn, and phenylacetylene was adjusted, and a compromise between reactivity and stereoselectivity was finally obtained. Similar reaction with aliphatic or aromatic ketones do not give any traces of product if they are conducted at −20 °C, even in the presence of the Salen ligand. The best conditions in the optimization were found when 4.5 equivalent of Me_2_Zn and 30 mol % of Salen were employed in the reaction. The Salen and the Me_2_Zn were mixed at rt for 1 hour before being to add the chloroketone at low temperature. The quantity of solvent was also important in order to enhance the enantiomeric excess. The reaction was performed without stirring, at −20 °C for 60 hours. 

**Table 2:** Stereoselective addition of phenylacetylene to 3-chlorobutanone promoted by (*R,R*)-Salen ligand.

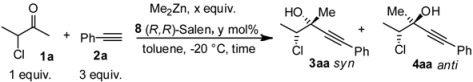								
Entry ^a^	Me_2_Zn	Salen	solvent	dr ^b^	T (h)	ee *anti* ^c^	ee *syn* ^c^	Yield ^d^
	(x equiv.)	(y mol %)	(mL)					
1	3	20	1	78:22	72	70	40	25
2	5	20	1	77:23	72	79	45	45
3	6	20	1	76:24	60	50	50	53
4	4.5	30	1.5	82:18	60	90	82	25
5	4.5	10	1	83:17	60	50	67	69

^a^ All the reactions were performed at −20 °C under nitrogen and stopped after the time indicated. ^b^ The diastereoisomeric ratio was evaluated on the crude reaction mixture by ^1^H-NMR. ^c^ The enantiomeric excess was evaluated by chiral HPLC analysis (see experimental part for the conditions) ^d^ Isolated yield (*syn* + *anti*) after chromatographic purification.

**Scheme 3:**
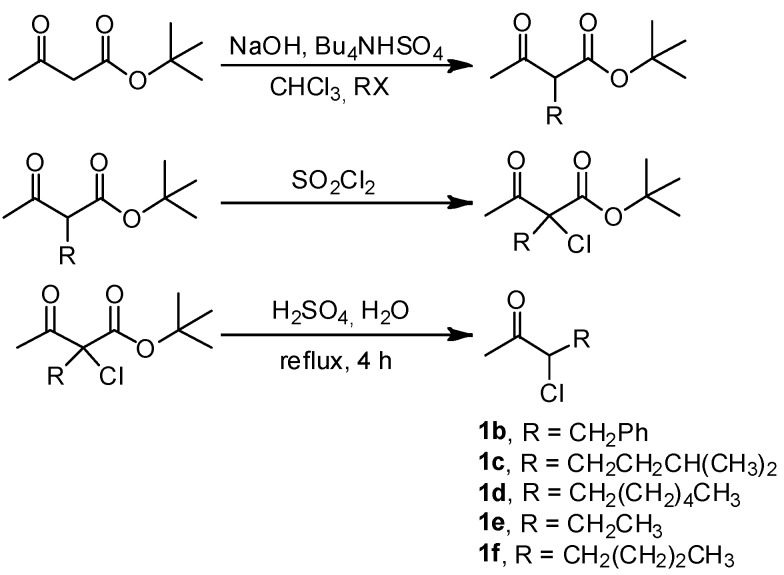
Preparation of chloroketones.

Delighted by the results obtained with 3-chlorobutanone, we decided to investigate the generality of our reaction, studying other different chloroketones. The substrates were prepared using a methodology developed by De Kimpe [23], illustrated in Scheme 3.

The corresponding ketones were prepared without difficulties in large scale, and were purified by distillation. The ketones **1b-f** was used in the reaction with phenylacetylene promoted by Me_2_Zn, by using the conditions optimized for the substrate **1a** and the results are reported in Table 3.

**Table 3:** Stereoselective addition of phenylacetylene to a series of α-chloroketones promoted by (*R,R*)-Salen ligand.

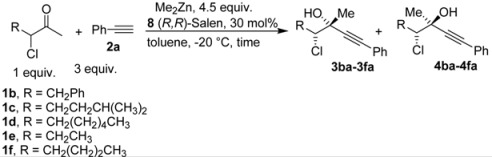						
Entry ^a^	Ketone	dr ^b^	T (h)	ee *anti* ^c^	ee *syn* ^c^	Yield ^d^ ( *syn* + *anti*)
1	**1a**	82:18	60	90	82	26
2	**1b**	73:27	120	85	65	35
3	**1c**	77:23	60	81	58	25
4	**1d**	78:22	60	82	55	18
5	**1e**	75:25	140	62	42	40
6	**1f**	80:20	60	83	57	17

^a^ All the reactions were performed at −20 °C under nitrogen and stopped after the time indicated. ^b^ The diastereoisomeric ratios were evaluated on the crude reaction mixture by ^1^H-NMR. ^c^ The enantiomeric excess was evaluated by chiral HPLC analysis (see experimental part for the conditions) ^d^ Isolated yield (*syn* + *anti*) after chromatographic purification.

**Scheme 4:**
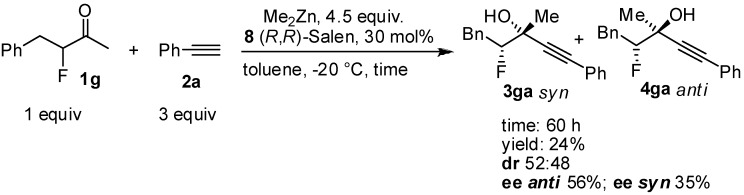
Stereoselective addition of phenylacetylene to the fluoroketone **1g**.

Generally, the yields of the reaction are quite moderate, because the reaction was stopped in order to obtain the highest enantiomeric excesses for the isolated adducts. The steric hindrance of the ketones does not seem significant in the controlling the enantiomeric excess. The reaction also works in the case of other haloketones, e.g. *α*.-fluoroketones, Scheme 4 Although was possible to perform the reaction with other haloketones, generally the results were inferior compared to the chloroketones. 

Concerning the possibility of using different substituted acetylenes in our reactions, we have briefly investigated the reaction employing alkyl and silyl substituted acetylenes under the general conditions developed for 3-chlorobutanone, and the data obtained are reported in Table 4. As is possible to evince by the data, the reactivity of the acetylenes **2b-d** was quite lower compared to phenylacetylene, and products were isolated in low yield and with minor levels of stereoselectivity.

**Table 4:** Stereoselective addition of substituted acetylenes to 3-chlorobutanone promoted by (*R*,*R*)-Salen ligand.

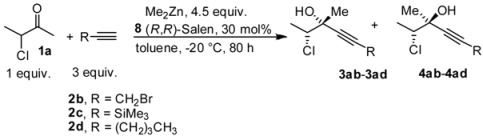						
Entry ^a^	Alkyne	dr ^b^	ee *anti* ^c^	ee *syn* ^c^	Yield ^d^ ( *syn* + *anti*)
1	HCCCH_2_Br, **2b**	64:36	66	53	25
2	HCCSiMe_3,_ **2c**	75:25	75	62	15
3	HCC(CH_2_)_3_CH_3_, **2d**	83:17	68	41	20

^a^ All the reactions were performed at −20 °C under nitrogen atmosphere, by adding Me_2_Zn (4 equiv) to Salen (30 mol%) at rt. Then the alkyne was added and the mixture was cooled to −25 °C. The ketone **1a** was added and the reaction kept without stirring for 80 h at −20 °C. ^b^ The diastereoisomeric ratios (*anti*: *syn*) were evaluated on the crude reaction mixture by ^1^H-NMR. ^c^ The enantiomeric excess was evaluated by chiral HPLC analysis (see experimental part for the conditions) ^d^ Isolated yield (*syn* + *anti*) after chromatographic purification.

**Scheme 5:**
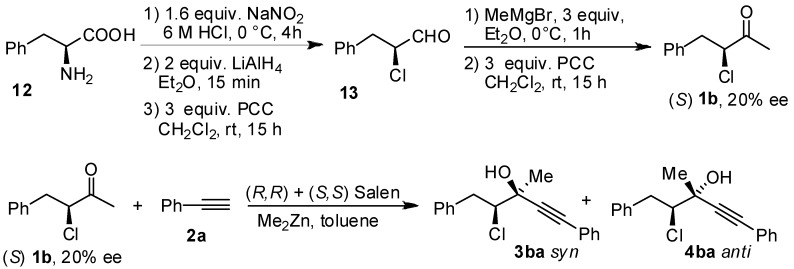
Assignment of absolute configuration of the *syn* and *anti* stereoisomers on the basis of diastereoselective addition to the enantioenriched (*S*)**-1b**.

We have established the absolute configuration of the products as indicated in Scheme 5. Enantiopure 4-phenyl-3-chloro-butan-2-one was prepared from the corresponding (*S*)-3-phenyl-2-chloropropanal following the procedure published by De Kimpe [24]. 

**Figure 1:**
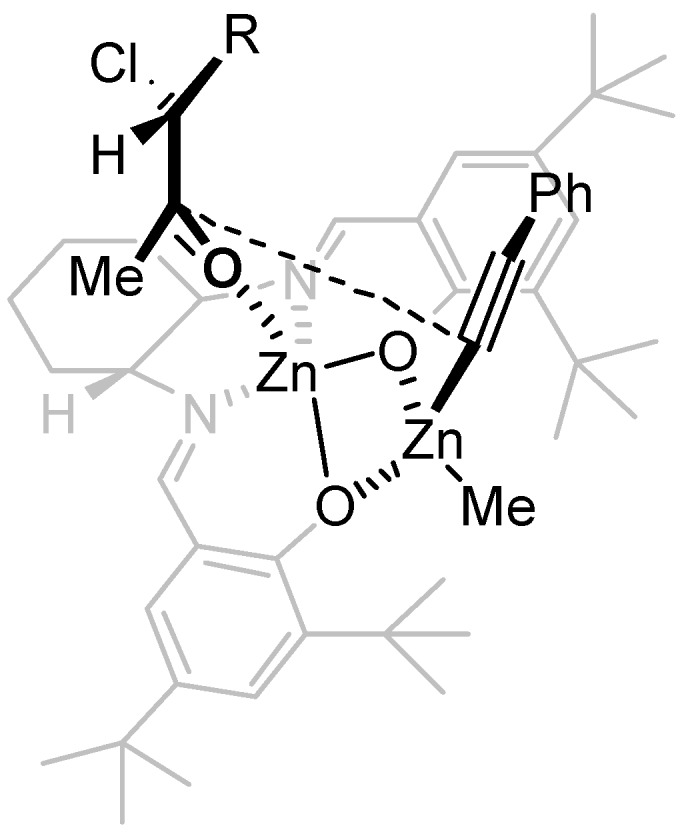
Stereoselective addition of phenylacetylene on the chloroketone.

The (*S*)-chloroaldehyde **13** was obtained in moderate yield from the aminoacid **12**, and used without purification in the subsequent steps. (*S*)-chloroaldehyde **11** was treated with MeMgBr at 0 °C, to give the corresponding secondary alcohol that was directly oxidized with PCC to the ketone **1b**. HPLC analysis performed on the chloroketone established that the (*S*)-chloroketone was obtained with poor enantiomeric excess of 20%, due to the racemization. Optically active chloroaldehydes have been used in the diastereoselective addition of organometallic reagents [25,26,27,28,29]. The addition of Grignard reagents to chloroaldehyde was reported to give good yield and moderate diastereoisomeric excess both in Et_2_O or in THF, and racemization of the aldehydes seems not to occur [30]. Perhaps, the racemization of our substrate is occurring during the oxidation step. Nevertheless, the enantiomeric excess obtained for the chloroketone **1b** was sufficient to assign the absolute configuration of the products obtained in the alkynylation reactions. The reaction with the chloroketone **1b** was performed in the presence of 20 mol% of a racemic mixture of (*R*,*R*) and (*S*,*S*)-Salen ligand (Scheme 5). AS in the case of the ketone **1a**, the diastereoisomeric ratio of products **3ba** and **4ba** obtained was 4:1 in favor of the *anti* diastereoisomer. The configuration of the stereogenic centers for all the diastereoisomers was assigned by comparison of the HPLC traces obtained for the racemic and for the (*S*)-chloroketone. The absolute configuration of the major diastereoisomer obtained in the reaction with (*R*,*R*)-Salen and racemic **1b** was 3*S*,4*R*. The absolute configuration for all the products was assigned by analogy, taking in consideration that for all the chloroketones the *anti* and *syn* products have similar HPLC retention times. Is worth adding that the absolute configuration obtained in the case of the chloroketone is opposite to that in the Salen mediated addition of phenylacetylene to aliphatic and aromatic ketones. In the case of aliphatic and aromatic ketones the (*R*,*R*)-Salen is inducing the formation of a new stereogenic tertiary alcohol of (*S*) configuration [31]. However, the reaction conditions for the alkynylation reactions in the case of the chloroketones are completely different from those of the alkynylations of aromatic and aliphatic ketones. In addition, the presence of the stereogenic center of the chiral chloroketone can induce a preferential coordination of one enantiomer of the chloroketone as depicted in the model of Figure 1. In order to study the possibility of kinetic resolution, two enantiomeric chloroketones **1b** were separated by chiral HPLC analysis. When the reaction was performed using 2 or more equivalents of chloroketone **1b**, the excess of chloroketone was isolated after the reaction performed in the presence of (*R*,*R*)-Salen ligand and it was analyzed by chiral HPLC. The ketone **1b** was isolated with no traces of enantioenrichment. In addition, when the optically active (*S*)**-1b** (20% ee) was treated with 2 equivalent of Me_2_Zn in toluene and the solution obtained was stirred at rt for two days, after quenching no reaction of Me_2_Zn with the choroketones took place. According to the HPLC analysis of the crude reaction mixture the (*S*) chloroketone undergoes no racemization in the presence of Me_2_Zn, as the enantiomeric excess of the chloroketones was unchanged. Therefore, the result obtained in the reaction of racemic chloroketones is not determined by a racemization of the chloroketones and the selective reaction of one enantiomer. The observed facial stereoselection could result from a preferential coordination of one enantiomer of the chloroketone to the Zn(Salen) complex, with this preferential stereoisomer reacting at a faster rate. It is worth adding that the analysis of the reaction is complicated by the fast background reaction, which has hampered any attempt to prove that the reaction was taking place via a kinetic resolution with preferential coordination of the (*R*) chloroketones. The fast background reaction is favored by excess of ketones, an excess that is necessary to investigate the kinetic resolution. For example, when the reaction was performed using 3 equiv of **1a** in the presence 1 equiv of phenylacetylene and 1.4 equiv. of Me_2_Zn, the corresponding adducts were isolated with 15% ee for the *anti* stereoisomer. Performing careful analysis and selecting different reaction times and concentrations, we were not able to measure any enrichment of the starting chloroketones **1b**. However, further studies are still necessary in order to explain and understand the results obtained in the stereoselective addition of phenylacetylene to haloketones, and work is in progress towards this objective.

## 3. Experimental

### 3.1. General

^1^H-NMR spectra were recorded on Varian Gemini 200 and Varian Mercury 400 spectrometers. Chemical shifts are reported in ppm from TMS with the solvent resonance as the internal standard (deuterochloroform: *δ* = 7.27 ppm). Data are reported as follows: chemical shift, multiplicity (s = singlet, d = duplet, t = triplet, q = quartet, bs = broad singlet, m = multiplet), coupling constants (Hz). ^13^C-NMR spectra were recorded on Varian Gemini 200 and Varian Mercury 400 spectrometers. Chemical shifts are reported in ppm from TMS with the solvent as the internal standard (deuterochloroform: *δ* = 77.0 ppm). GC-MS spectra were taken by EI ionization at 70 eV on a Hewlett-Packard 5971 with GC injection. They are reported as: *m/z* (rel. intense). LC-electrospray ionization mass spectra were obtained with Agilent Technologies MSD1100 single-quadrupole mass spectrometer. Chromatographic purification was done with 240–400 mesh silica gel. Determination of enantiomeric excesses were performed on an Agilent Technologies 1200 instrument equipped with a variable wave-length UV detector, using Daicel Chiralpak columns (0.46 cm I.D. × 25 cm) and HPLC grade isopropanol and *n*-hexane were used as the eluting solvents. Melting points were determined with a Bibby Stuart Scientific SMP 3 Melting Point Apparatus and are not corrected. All reactions were carried out under inert gas and anhydrous conditions. Anhydrous solvents were supplied by Aldrich in Sureseal^®^ bottles and used avoiding purification. Me_2_Zn 2M in toluene was supplied in Aldrich Sureseal^®^ bottles and used as received. (*R*,*R*)-Salen is commercially available from Aldrich. Phenyacetylene was purchased by Aldrich and used as received. 3-Chlorobutanone is commercially available and was used after distillation. The chloroketones **1b**-**1f** were prepared according to the literature procedure described by De Kimpe [23], and were obtained in 16–20% yield (three reactions). The fluoroketone **1g** was obtained as reported in literature [32].

*3-Chloro-4-phenylbutan-2-one* (**1b**). C_10_H_11_ClO MW = 182.65. ^1^H-NMR (CDCl_3_, 300 MHz) δ: 2.35 (s, 3H); 3.11 (dd, 1H, *J* = 8.1, 14.4 Hz); 3.37 (dd, 1H, *J* = 6.3, 14.4 Hz); 4.44 (dd, 1H, *J* = 6.3, 8.1 Hz). 7.4–7.2 (m, 5H). ^13^C-NMR (CDCl_3_, 75 MHz) δ: 26.8; 39.8; 63.8; 127.2; 128.2; 128.6; 129.3. 


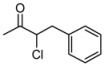


*3-Chloro-6-methyleptan-2-one* (**1c)**. C_8_H_15_ClO MW = 162.08. ^1^H-NMR (CDCl_3_, 300 MHz) δ: 0.92 (d, 3H, *J* = 6.6 Hz); 0.97 (d, 3H, *J* = 6.6 Hz); 1.8–1.71 (m, 5H); 2.32 (s, 3H); 4.22 (dd, 1H, *J* = 6.0, 9.0 Hz). ^13^C-NMR (CDCl_3_, 75 MHz) δ: 21.2; 22.7, 25.0, 25.6; 62.7, 203.5.


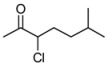


*3-Chlorononan-2-one* (**1d**). C_9_H_17_ClO MW = 148.63. ^1^H-NMR (CDCl_3_, 300 MHz) δ: 0.88 (m, 3H); 2.0–1.20 (m, 10H); 2.31, (s, 3H); 4.17 (dd, 1H, *J* = 5.4, 8.1 Hz). ^13^C-NMR (CDCl_3_, 75 MHz) δ: 13.9; 22.4; 25.8; 25.9; 28.5; 28.9; 31.4; 33.7; 64.3; 203.4.


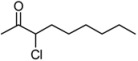


*3-Chloropentan-2-one* (**1e**). C_5_H_9_ClO MW = 120.58. ^1^H-NMR (CDCl_3_, 300 MHz) δ: 1.02 (t, 3H, *J* = 7.8 Hz); 2.1–1.8 (m, 2H); 2.31 (s, 3H); 4.12 (dd, 1H, *J* = 5.4, 7.8 Hz). ^13^C-NMR (CDCl_3_, 75 MHz) δ: 10.5; 25.9; 27.2; 65.7; 203.3.


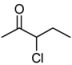


*3-Chloroheptan-2-one* (**1f**). C_7_H_13_ClO MW = 148.63. ^1^H-NMR (CDCl_3_, 300 MHz) δ: 0.92 (t, 3H, *J* = 6.6 Hz); 1.40–1.31 (m, 4H); 2.0–1.80 (m, 2H); 2.31 (s, 3H); 4.16 (dd, 1H, *J* = 5.7, 8.1 Hz). ^13^C-NMR (CDCl_3_, 75 MHz) δ: 13.8; 22.0; 25.8; 28.1; 33.5; 64.3; 203.5.


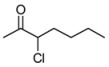


### 3.2. Addition of Alkynes to Chloroketones

*General procedure*: In a flask under nitrogen containing a solution of (*R*,*R*)Salen (0.075 mg, 0.135 mmol) in toluene (1 mL), a 2 M solution of Me_2_Zn is added under stirring (1 mL, 2.025 mmol). The mixture is stirred 10 min at rt, then phenylacetylene (0.15 mL, 1.35 mmol) was added. The mixture was stirred 1 h at rt, then the solution was cooled to –25 °C. Chloroketone (0.45 mmol) was added, and the mixture was kept at –20 °C without stirring for 60 h. The reaction was quenched with water at –20 °C, then the reaction was diluted with Et_2_O. The organic phase (yellow) was separated and the aqueous phase was extracted with Et_2_O. The organic phases were reunited, evaporated under reduce pressure and purified by chromatography.

*4-Chloro-3-methyl-1-phenylpent-1-yn-3-ol* **(3aa/4aa)**. C_12_H_13_ClO MW= 208.68. ^1^H-NMR (CDCl_3_, 200 MHz) δ: 1.67 (maj, s, 3H); 1.68 (min, s, 3H); 1.72 (d, 3H, *J* = 6.6 Hz); 1.61 (min, brs, 1H); 2.86 (maj, brs, 1H); 4.16 (maj, q, 1H, *J* = 6.6 Hz); 4.23 (min, q, 1H, *J* = 7.0 Hz); 7.35–7.30 (m, 3H); 7.50–7.46 (m, 2H). ^13^C-NMR (CDCl_3_, 50 MHz) δ: 20.0 (min); 20.7 (maj); 25.5 (min); 26.7 (maj); 65.2 (min); 68.8 (maj); 71.3 (min); 71.6 (maj); 84.8 (min); 85.1 (maj); 88.7 (maj); 89.7 (min); 122.2; 128.2; 128.6; 131.7. The ee was determined by HPLC analysis (Daicel Chiralcel OD column: hexane/*i*-PrOH 98.5:1.5, flow rate 0.50 mL/min, 27 °C, λ = 214, 250 nm: *anti* diasteroisomer (**4aa**) *τ_major_* = 23.13 min, *τ_minor_ =* 41.21 min; *syn* diasteroisomer (**3aa**) *τ_major_* = 28.58 min., *τ_minor_ =* 45.83 min. *anti*:*syn* 82:18. ee *anti* 85%. ee *syn* 65%. GCMS: 15.07 (maj); 15.16 (min). HMRS calcd for C_12_H_13_ClO: 208.0655; found 208.0659.


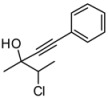


*2,3-Dimethyl-2-phenylethynyloxirane* (**10/11**). C_12_H_13_ClO MW = 172.22. ^1^H-NMR (CDCl_3_, 200 MHz) δ: 1.38 (maj, d, 3H, *J* = 5.6 Hz); 1.52 (min, d, 3H, *J* = 5.2 Hz); 1.61 (maj, s, 3H); 1.65 (min, s, 3H); 3.08 (min, q, *J* = 5.2 Hz); 3.39 (maj, q, 1H, *J* = 5.4 Hz); 7.35–7.30 (m, 3H); 7.50–7.46 (m, 2H). ^13^C-NMR (CDCl_3_, 50 MHz) δ: 13.7 (maj); 15.6 (min); 18.3 (maj); 23.3 (min); 51.2 (maj); 53.2 (min); 60.9 (maj); 61.4 (min); 81.6; 89.9; 122.2; 128.2; 128.5; 131.8.


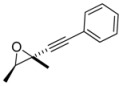


*4-Chloro-3-methyl-1,5-diphenylpent-1-yn-3-ol* (**3ba/4ba**). C_18_H_17_ClO MW = 284.78. ^1^H-NMR (CDCl_3_, 300 MHz) δ: 1.68 (s, 3H); 2.85 (min, dd, 1H, *J* = 11.1, 14.4 Hz); 2.99 (maj, dd, 1H, *J* = 10.8, 14.4 Hz); 3.55-3.41 (m, 1H); 4.09 (min, dd, 1H, *J* = 2.4, 11.1 Hz); 4.19 (maj, dd, 1H, *J* = 2.7, 10.8 Hz); 7.60–7.20 (m, 10H). ^13^C-NMR (CDCl_3_, 75 MHz) δ: 26.2 (maj); 26.8 (min); 27.3; 39.8 (min); 40.1 (maj); 71.2 (min); 71.3 (maj); 71.4 (min); 72.7 (maj); 85.5 (min); 85.6 (maj); 89.1 (maj); 89.4 (min); 122.1; 126.8; 128.3; 128.4; 128.7; 129.3; 131.8; 137.9 (maj); 138.0 (min). The ee was determined by HPLC analysis Daicel Chiralcel OD column: hexane/*i*-PrOH 97.5:2.5, flow rate 0.50 mL/min, 27 °C, λ = 214, 250 nm: *anti* diasteroisomer (**4ba**) *τ_major_* = 21.97 min, *τ_minor_ =* 30.96 min; *syn* diasteroisomer (**3ba**) *τ_major_* = 23.48 min., *τ_minor_ =* 32.14 min. a*nti*:*syn* 73:27. ee *anti* 85%. ee *syn* 65%. HMRS calcd for C_18_H_17_ClO: 284.0978; found 284.0982. Daicel Chiralcel ODH column: hexane/*i*-PrOH 97.5:2.5, flow rate 0.50 mL/min, 27 °C, λ = 214, 250 nm: *anti* diasteroisomer *τ_major_* = 31.79 min, *τ_minor_ =* 43.47 min; *syn* diasteroisomer *τ_major_* = 35.61 min, *τ_minor_ =* 50.09 min.


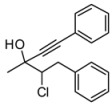


*4-Chloro-3,7-dimethyl-1-phenyloct-1-yn-3-ol* (**3ca/4ca**). C_15_H_19_ClO MW = 264.13. ^1^H-NMR (CDCl_3_, 300 MHz) δ: 0.88 (d, 3H, *J* = 6.6 Hz); 0.93 (d, 3H, *J* = 6.6 Hz); 1.59 (min, s, 3H); 1.6 (maj, s, 3H); 2.0–1.40 (m, 5H); 2.43 (min, brs, 1H); 2.8 (maj, brs, 1H); 3.95 (maj, dd, 1H, *J* = 2.1, 11.4 Hz); 4.06 (min, dd, 1H, *J* = 4.8, 7.5 Hz); 7.30–7.20 (m, 3H); 7.41–7.35 (m, 2H). ^13^C-NMR (CDCl_3_, 75 MHz) δ: 20.6; 23.6 (maj); 25.3 (maj); 25.6 (min); 27.0 (min); 41.9 (maj); 42.4 (min); 69.2 (maj); 70.8 (maj); 71.3 (min); 71.4 (min); 85.3 (maj); 86.1 (min); 89.3 (min); 89.9 (maj); 122.2; 128.2; 128.6; 131.8. The ee was determined by HPLC analysis Daicel Chiralcel OD column: hexane/*i*-PrOH 99:1, flow rate 0.50 mL/min, 27 °C, λ = 214, 250 nm: *anti* diasteroisomer (**4ca**) *τ_major _*= 17.58 min, *τ_minor_ =* 47.78 min; *syn* diasteroisomer (**3ca**) *τ_major_* = 21.29 min, *τ_minor_ =* 50.02 min. *anti*:*syn* 77:23. ee *anti* 81%. ee *syn* 58%. HMRS calcd for C_15_H_19_ClO: 264.1281; found 264.1290.


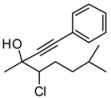


*4-Chloro-3-methyl-1-phenyldec-1-yn-3-ol* (**3da/4da**). C_17_H_23_ClO MW = 278.82. ^1^H-NMR (CDCl_3_, 300 MHz) δ: 0.82 (t, 3H, *J* = 7.2 Hz); 1.4–1.20 (m, 8H); 1.60 (s, 3H); 2.20–2.0 (m, 2H); 2.42 (maj, brs, 1H); 2.8 (min, brs, 1H); 3.88 (min, dd, 1H, *J* = 2.1, 11.1 Hz); 3.98 (maj, dd, 1H, *J* = 2.4, 10.8 Hz); 7.30–7.20 (m, 3H); 7.41–7.35 (m, 2H). ^13^C-NMR (CDCl_3_, 75 MHz) δ: 14.0 (maj); 15.2 (min); 22.5; 25.7; 27.0; 28.6; 31.6; 30.6 (maj); 33.5 (min); 71.2 (maj); 71.3 (min); 84.6 (maj); 84.8 (min); 89.3 (min); 89.9 (maj); 122.2; 128.2; 128.6; 131.7. The ee was determined by HPLC analysis Daicel Chiralcel OD column: hexane/*i*-PrOH 99.5:0.5, flow rate 0.50 mL/min, 27 °C, λ = 214, 250 nm: *anti* diasteroisomer (**4da**) *τ_major_* = 23.20 min, *τ_minor_ =* 65.06 min; *syn* diasteroisomer (**3da**) *τ_major_* = 30.40 min, *τ_minor_ =* 75.63 min. *anti*:*syn* 78:22. ee *anti* 82%. ee *syn* 55%. HMRS calcd for C_17_H_23_ClO: 278.1437; found 278.1441.


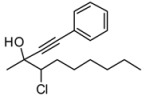


*4-Chloro-3-methyl-1-phenylhex-1-yn-3-ol* (**3ea/4ea**). C_13_H_15_ClO MW = 222.71. ^1^H-NMR (CDCl_3_, 300 MHz) δ: 1.16 (min, t, 3H, *J* = 6.9 Hz); 1.51 (min, t, 2H, *J* = 6.9 Hz); 1.59 (min, s, 3H); 1.68 (maj, s, 3H); 2.26–2.08 (m, 2H); 3.88 (min, dd, 1H, *J* = 2.1, 11.1 Hz); 3.97 (maj, dd, 1H, *J* = 2.1, 11.4 Hz); 7.35–7.22 (m, 3H); 7.45–7.30 (m, 2H). ^13^C-NMR (CDCl_3_, 75 MHz) δ: 12.0; 25.8 (maj); 26.4 (maj); 26.9 (min); 27.0 (min); 71.3 (min); 73.1; 74.3 (maj); 84.9; 89.3 (min); 89.8 (maj); 122.2; 128.2; 128.5; 131.7. GC: 16.40 (min); 16.52 (maj). The ee was determined by HPLC analysis Daicel Chiralcel OD column: hexane/*i*-PrOH 98:2, flow rate 0.50 mL/min, 27 °C, λ = 214, 250 nm: *anti* diasteroisomer (**4ea**) *τ_major_* = 25.53 min, *τ_minor_ =* 51.00 min; *syn* diasteroisomer (**3ea**) *τ_major_* = 34.73 min, *τ_minor _=* 62.74 min. *anti*:*syn* 75:25. ee *anti* 62%. ee *syn* 42%. GCMS: 15.07 (maj); 15.16 (min). HMRS calcd for C_13_H_15_ClO: 208.0811; found 208.0815.


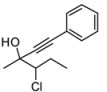


*4-Chloro-3-methyl-1-phenyloct-1-yn-3-ol* (**3fa/4fa**). C_15_H_19_ClO MW = 250.76. ^1^H-NMR (CDCl_3_, 300 MHz) δ: 0.98 (t, 3H, *J* = 7.2 Hz); 2.0–1.20 (m, 6H); 1.70 (s, 3H); 2.50 (min, brs, 1H); 2.87 (maj, brs, 1H); 3.85 (maj, dd, 1H, *J* = 2.4, 11.1 Hz); 3.98 (min, m, 1H); 7.35–7.22 (m, 3H); 7.45–7.30 (m, 2H).^13^C-NMR (CDCl_3_, 75 MHz) δ: 13.9; 22.0; 25.7 (min); 27.0 (maj); 29.2; 32.7 (min); 33.3 (maj); 71.1 (min); 72.7 (maj); 85.0; 89.3 (maj); 89.6 (min); 122.3; 128.3; 128.5; 131.7. GC: 18.9 (maj); 19.0 (min). The ee was determined by HPLC analysis Daicel Chiralcel OD column: hexane/*i*-PrOH 98:2, flow rate 0.50 mL/min, 27 °C, λ = 214, 250 nm: *anti* diasteroisomer (**4fa**) *τ_major_* = 15.77 min, *τ_minor_ =* 26.90 min; *syn* diasteroisomer **(3fa**) *τ_major_* = 18.16 min, *τ_minor_ =* 28.48 min. *anti*:*syn* 80:20. ee *anti* 83%. ee *syn* 57%. GCMS: 15.07 (maj); 15.16 (min). HMRS calcd for C_13_H_15_ClO: 208.0811; found 208.0815.


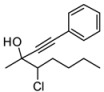


*2-Fluoro-3-methyl-1,5-diphenylpent-4-yn-3-ol* (**3ga/4ga**). C_18_H_17_FO MW = 268.33. ^1^H-NMR (CDCl_3_, 300 MHz) δ: 1.62 (d, 3H, *J* = 9.0 Hz); 3.25–2.96 (m, 2H); 4.67–4.45 (m, 1H); 7.28–7.20 (m, 8H); 7.45–7.38 (m, 2H); ^13^C-NMR (CDCl_3_, 75 MHz) δ: 25.9 (d, *J* = 123 Hz); 36.7 (min, d, *J* = 85 Hz); 36.9 (maj, d, *J* = 85 Hz); 70.2 (maj, d, *J* = 83 Hz); 70.0 (min, d, *J* = 83 Hz); 97.9 (min, *J* = 725 Hz); 98.5 (maj, d, *J* = 725 Hz): 122.0; 128.2; 128.5; 128.7; 129.2; 131.7; 137.34; The ee was determined by HPLC analysis Daicel Chiralcel OD column: hexane/*i*-PrOH 97:3, flow rate 0.50 mL/min, 27 °C, λ = 214, 250 nm: *anti* diasteroisomer (**4ga**) *τ_major_* = 30.23 min, *τ_minor_ =* 39.07 min; *syn* diasteroisomer (**3ga**) *τ_major_* = 33.45 min, *τ_minor_ =* 40.72 min. *anti:syn* 52:48. ee *anti* 56%. ee *syn* 35%. HMRS calcd for C_18_H_17_FO: 268.1273; found 268.1268.


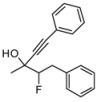


*6-Bromo-2-chloro-3-methyl-1-phenylhex-4-yn-3-ol* (**3ab/4ab**). C_13_H_14_BrClO MW= 301.61. ^1^H-NMR (CDCl_3_, 300 MHz) δ: 1.59 (min, s, 3H); 1.69 (maj, s, 3H); 2.6 (brs, 1H); 3.60–2.75 (m, 1H); 3.55–3.44 (m, 1H); 4.01 (s, 2H); 4.13 (t, 1H, *J* = 11.1 Hz); 7.40–7.20 (m, 5H).^13^C-NMR (CDCl_3_, 75 MHz) δ: 13.7; 25.9 (maj); 27.0 (min); 36.6 (maj); 37.0 (min); 71.0 (maj); 71.1 (min); 72.4; 80.8 (maj); 80.5 (min); 86.8 (min); 87.0 (maj); 126.8; 128.5; 129.3; 137.7 (min); 137.8 (maj). The ee was determined by HPLC analysis Daicel Chiralcel OD column: hexane/*i*-PrOH 98:2, flow rate 0.60 mL/min, 27 °C, λ = 214, 250 nm: *anti* diasteroisomer (**4ab**) *τ_major_* = 38.36 min, *τ_minor_ =* 56.42 min; *syn* diasteroisomer (**3ab**) *τ_major_* = 44.62 min, *τ_minor_ =* 65.56 min. *anti*:*syn* 64:36. ee *anti* 66%. ee *syn* 53%. HMRS calcd for C_13_H_14_BrClO: 299.9917; found 299.9923.


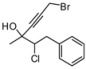


*4-Chloro-3-methyl-5-phenyl-1-(trimethylsilyl)pent-1-yn-3-ol* (**3ac/4ac**). C_15_H_21_ClOSi MW = 280.87. ^1^H-NMR (CDCl_3_, 300 MHz) δ: 0.24 (s, 9H); 1.68 (s, 3H); 3.01 (dd, 1H, *J* = 10.8, 14.1 Hz); 3.52 (d, 1H, *J* = 14.1 Hz); 4.08 (maj, 1H, dd, *J* = 2.4, 10.8 Hz); 4.18 (min, m, 1H); 7.40–7.20 (m, 5H).^13^C-NMR (CDCl_3_, 75 MHz) δ: −0.38 (min); −0.18 (maj); 27.4, 40.1; 70.9 (maj); 71.4 (min); 72.4; 90.5; 105.5; 126.8; 128.4; 129.3; 138.0. The ee was determined by HPLC analysis Daicel Chiralcel OD column: hexane/*i*-PrOH 99.6:0.4, flow rate 0.50 mL/min, 27 °C, λ = 214, 250 nm: *anti* diasteroisomer (**4ac**) *τ_major _*= 28.53 min, *τ_minor_ =* 27.34 min; *syn* diasteroisomer (**3ac**) *τ_major _*= 34.77 min, *τ_minor _=* 38.21 min. *anti*:*syn* 75:25. ee *anti* 75%. ee *syn* 62%. HMRS calcd for C_15_H_21_ClOSi: 280.1050; found 280.1054.


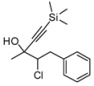


***2-****Chloro-3-methyl-1-phenylnon-4-yn-3-ol* (**3ad/4ad**). C_16_H_21_ClO MW = 301.61. ^1^H-NMR (CDCl_3_, 300 MHz) δ: 0.94 (t, 3H, *J* = 7.2 Hz); 1.60–1.40 (m, 4H); 1.63 (min, s, 3H); 1.65 (maj, s, 3H); 2.28 (t, 2H, *J* = 6.9 Hz); 2.43 (min, brs, 1H); 2.60 (maj, brs, 1H); 2.85 (min, dd, 1H, *J* = 11.1, 14.4 Hz); 2.98 (maj, dd, *J* = 10.8, 14.7 Hz); 3.56–3.46 (m, 1H); 4.07 (maj, dd, 1H, *J* = 2.7, 11.1 Hz); 4.12 (min, dd, *J* = 2.4, 10.8 Hz); 7.40–7.20(m, 5H). ^13^C-NMR (CDCl_3_, 75 MHz) δ: 13.5; 18.3; 21.9; 26.2 (min); 26.9 (maj); 30.6; 39.9 (min); 40.1 (maj); 70.8 (maj); 71.0 (min); 80.4 (maj); 80.7 (min); 126.7; 128.4; 129.2; 138.2. The ee was determined by HPLC analysis Daicel Chiralcel OD column: hexane/*i*-PrOH 99.6:0.4, flow rate 0.50 mL/min, 27 °C, λ = 214, 250 nm: *anti* diasteroisomer (**4ad**) *τ_major_* = 45.71 min, *τ_minor_ =* 49.73 min; *syn* diasteroisomer (**3ad**) *τ_major _*= 59.01 min, *τ_minor _=* 75.52 min. *anti*:*syn* 83:17. ee *anti* 68%. ee *syn* 41%. HMRS calcd for C_13_H_14_BrClO: 299.9917; found 299.9923.


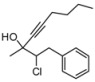


## 4. Conclusions

In conclusion, we have presented the first diastereoselective and enantioselective addition of phenylacetylene to chiral racemic chloroketones. The reaction provides access to highly functionalized products and the adducts were obtained in low yield and good stereoselection. The reactivity of chloroketones in the presence of chiral zinc catalyst can be explored with other zinc reagents taking advantage of the enhanced reactivity. Formation of quaternary and tertiary stereogenic centers from racemic chloroketones will be the subject of other studies from our laboratory. 
